# Clinical pain and functional network topology in Parkinson’s disease: a resting-state fMRI study

**DOI:** 10.1007/s00702-018-1916-y

**Published:** 2018-08-21

**Authors:** Gwenda Engels, Brónagh McCoy, Annemarie Vlaar, Jan Theeuwes, Henry Weinstein, Erik Scherder, Linda Douw

**Affiliations:** 1 Department of Clinical, Neuro- and Developmental Psychology, Faculty of Behavior- and Movement Sciences, VU University, Van der Boechorststraat 1, 1081 BT Amsterdam, The Netherlands; 2 Department of Experimental and Applied Psychology & Institute of Brain and Behavior Amsterdam, Faculty of Behavior- and Movement Sciences, VU University, Van der Boechorststraat 1, Amsterdam, The Netherlands; 3 Department of Neurology, OLVG West, Amsterdam, The Netherlands; 4 Department of Anatomy and Neurosciences, VU University Medical Center, De Boelelaan 1117, 1081 HV Amsterdam, The Netherlands; 5 Department of Radiology, Athinoula A. Martinos Center for Biomedical Imaging, Massachusetts General Hospital, 149 13th St, Charlestown, MA USA

**Keywords:** Pain, Parkinson’s disease, Functional network topology, Resting-state fMRI

## Abstract

Pain is an important non-motor symptom in Parkinson’s disease (PD), but its underlying pathophysiological mechanisms are still unclear. Research has shown that functional connectivity during the resting-state may be involved in persistent pain in PD. In the present cross-sectional study, 24 PD patients (both during on and off medication phase) and 27 controls participated. We assessed pain with the colored analogue scale and the McGill pain questionnaire. We examined a possible pathophysiological mechanism with resting-state fMRI using functional network topology, i.e., the architecture of functional connections. We took betweenness centrality (BC) to assess hubness, and global efficiency (GE) to assess integration of the network. We aimed to (1) assess the differences between PD patients and controls with respect to pain and resting-state network topology, and (2) investigate how resting-state network topology (BC and GE) is associated with clinical pain in both PD patients and controls. Results show that PD patients experienced more pain than controls. GE of the whole brain was higher in PD patients (on as well as off medication) compared to healthy controls. GE of the specialized pain network was also higher in PD patients compared to controls, but only when patients were on medication. BC of the pain network was lower in PD patients off medication compared to controls. We found a positive association between pain and GE of the pain network in PD patients off medication. For healthy controls, a negative association was found between pain and GE of the pain network, and also between pain and BC of the pain network. Our results suggest that functional network topology differs between PD patients and healthy controls, and that this topology can be used to investigate the underlying neural mechanisms of pain symptoms in PD.

## Introduction

Parkinson’s disease (PD) was for a long time mainly considered a motor disease, with rigidity, bradykinesia and resting tremor as its cardinal symptoms. In recent decades, however, there has been growing interest in the non-motor symptoms of PD (Postuma et al. 2015), which may be equally or more incapacitating than the motor symptoms themselves (Chaudhuri et al. 2006). Pain is one such non-motor symptom that is present in about two-thirds of PD patients (Defazio et al. 2008; Broen et al. 2012), and puts an additional burden on patients’ quality of life (Quittenbaum and Grahn 2004; Shibley et al. 2008; Buhmann et al. 2017). It has been linked to several clinical factors, such as disease progression (Mylius et al. 2011), dopaminergic fluctuation (Brefel-Courbon et al. 2005; Nègre-pagès et al. 2008; Silva et al. 2008), depressed mood, and dyskinesias (Rodríguez-Violante 2017).

During the past few decades, possible neural substrates of pain have been studied extensively, resulting in a potential network of connected brain areas that is thought to underlie the processing and experience of pain (Tracey and Mantyh 2007). There is no definite consensus on all areas involved in such a pain network; nonetheless, pain-related regions consistently found across studies include the thalamus, anterior cingulate cortex (ACC), posterior and anterior insula, amygdala, prefrontal cortex (PFC), secondary somatosensory cortex (SII) and the periaqueductal grey (PAG) (Scherder et al. 2003). Wager and colleagues employed a machine-learning-based regression technique (LASSO-PCR) to identify such a network, attempted to predict the presence of physical pain (Wager et al. 2013). The result, a so-called neural pain signature (NPS), was found to predict physical pain with high sensitivity and high specificity. Many of the areas involved in this NPS are congruent with areas previously associated with pain, such as the insula, ACC, PFC, thalamus and SII (Tracey and Mantyh 2007).

Connectivity analysis of the resting-state, during which no specific task is performed, is based on patterns of co-activation of brain regions, providing insight into how the brain is functionally connected. The characteristics of such resting-state connectivity can be employed to study clinically relevant symptoms, such as cognitive functioning in PD (Olde Dubbelink et al. 2014) or pain in chronic back-pain patients (Tagliazucchi et al. 2010). Using magnetoencephalography (MEG), an increase in functional connectivity in PD was found to be associated with the duration and severity of the disease (Stoffers et al. 2008). A recent MRI study, investigating both structural and functional mechanisms of persistent pain in PD, found that resting-state connectivity between the right nucleus accumbens and the left hippocampus was reduced in PD patients with pain, compared to PD patients without pain (Polli et al. 2016; Antonini et al. 2018).

In addition to studying increases and decreases of functional connectivity, modern network science has provided an alternative method of investigating the brain, namely by studying specific characteristics of complex networks. Here, the brain (network) is comprised of brain regions (nodes), which have connections of varying strength between them (edges). In functional connectivity, these edges are based on the extent of co-activation of pairs of brain regions. A consensus in modern network science is that the brain is a cost-effective small-world network, which is both locally integrated (i.e., local clusters of connected nodes), and well connected on a global scale (i.e., connected nodes over longer distances) (see Stam for a review 2014). A number of measures of network topology, i.e., the architecture of connections, have been proposed (for an overview see Rubinov and Sporns 2010). Investigating the brain via functional network topology, as opposed to standard activation or simple functional connectivity measures, can provide further insight into the pathological mechanism of pain in PD.

A large body of literature in this field has examined the default mode network (DMN) (Raichle et al. 2001). Alterations of the DMN and its relationship with cognitive functioning have also been linked to disease (e.g., Alzheimer’s disease, epilepsy) (Broyd et al. 2009; Anticevic et al. 2012). Previous research has shown deviant functioning of the DMN at an early stage of PD (van Eimeren et al. 2009; Rektorova 2014), as well as in chronic pain patients (Baliki et al. 2014).

In this study, we investigated how both clinical pain and functional network topology [whole brain, the pain network (NPS) and DMN] differ between PD patients and controls. Furthermore, we examined the relationship between the amount of clinical pain and functional pain network topology during resting-state within each group. We hypothesized that subjective pain is related to functional topology of the pain network both in PD patients and in controls. Since dopamine is thought to affect both pain (Jääskeläinen et al. 2001) and resting-state functional connectivity (Kelly et al. 2009), PD patients were assessed both during an ON phase, in which dopaminergic medication was taken as usual, as well as an OFF phase, in which dopamine levels were low. We hypothesized differences between the ON and OFF phase with respect to both pain and resting-state functional connectivity.

## Materials and methods

For healthy controls, inclusion criteria were (1) aged 40–75 years, (2) provision of written informed consent, (3) normal or corrected-to-normal vision, and additionally for patients, (4) a diagnosis of PD following UK Brain Bank criteria. Exclusion criteria for all participants were (1) current use of psychotropic medication other than levodopa, dopamine-agonists or other Parkinson-medication, (2) major somatic disorder, (3) current psychiatric diagnosis as established by a psychiatrist, (4) presence of dementia, history of stroke or other neurological diseases (as established by neurologist). An additional screening for dementia was performed using the montreal cognitive assessment (MoCA), a screening tool for cognitive dysfunction (Nasreddine et al. 2005), with a cutoff for dementia according to Biundo and colleagues (2014). Patients were recruited through outpatient clinics. Healthy controls were recruited through advertisement in local newspapers, online advertisement and through participating patients (e.g., spouses, relatives, etc.).

### Procedure

The study was approved by the medical ethical committee of the VU Medical Center, Amsterdam. Informed consent was obtained from all individual participants included in the study. All methods were carried out accordance with relevant guidelines and regulations.

This study was part of a larger cross-sectional case–control study investigating visual attention, reward and pain in PD. Study size was based on expected learning and attention differences between groups. Note that only procedures concerning this project will be described. Patients visited the hospital twice: on the first visit, the MoCA was administered, and the questionnaires were handed in (filled out just prior to visit). During the second and third visit, the MRI was performed in either the ON or OFF phase. The same procedure was completed for the controls, with the exception that they underwent only one MRI session. The MRI was planned in the same week as the clinical assessment in almost all patients, but as a rule no later than 60 days after the clinical assessment. For the MRI, patients were invited to the hospital in the afternoon for the ON phase, and in the morning for the OFF phase. ON and OFF phase as the first or second MRI session was counter-balanced across participants. The OFF phase was defined as at least 12 h of dopaminergic medication overnight withdrawal. One patient took their medication 8.5 h before the resting-state scan to relieve symptoms.

### Pain

Here, we define clinical pain as naturally occurring pain that is not experimentally induced, regardless of origin or type. Ultimately, all pain processing is a neurophysiological phenomenon, irrespective of origin (Garland 2012). Pain was measured during the ON and OFF phase by means of the colored analogue scale (CAS) for intensity as well as for pain affect: subjects were asked to indicate the intensity of their pain (CAS intensity) and how much they were bothered by their pain (CAS affect) both on a scale ranging from ‘None’ (light pink, 0) to ‘Maximal’ (dark red, 100) (McGrath et al. 1996). The Dutch version of the McGill Pain Questionnaire was administered during the first visit to inquire about pain during the previous month (Melzack 1975). We utilized the total score on the number of words chosen (NWC) part of the McGill pain questionnaire. The NWC consists of three major classes of pain descriptors, which were used by the subjects to specify their pain experience. These classes are of sensory, affective or evaluative nature (van der Kloot et al. 1995). Total score on the NWC was used as score for each subject’s clinical pain experience.

### MRI

Imaging data were collected with a 3T GE Signa HDxT (General Electric, Milwaukee, WI, USA) at the VU University Medical Center (Amsterdam, The Netherlands). Structural images were acquired with a 3D T1-weighted MP RAGE sequence with the following acquisition parameters: voxel size = 1 mm isotropic, 176 slices, 256 × 256 matrix, repetition time (TR) = 8.2 ms, echo time (TE) = 3.2 ms, flip angle (FA) = 12°, inversion time (TI) = 450 ms. Resting-state data were acquired using a T2*-weighted echo-planar functional scan: number of volumes = 202, 42 slices, slice thickness = 3.2 mm, matrix size = 64 × 64, TR = 2150 ms, TE = 35 ms, FA = 80°, field of view = 240 mm, total duration 7:12 min, voxel size was 3 mm with 0.3 mm spacing. For the resting-state scan, subjects were instructed to close their eyes, lie still and avoid falling asleep. Participants’ heads were immobilized using foam pads to reduce motion artifacts.

### Processing of fMRI data

Data were analyzed using FSL FMRIB software library v5.0.9 (Jenkinson et al. 2012) and custom-built scripts in bash and Matlab, version 2015a (Mathworks, Natick, MA, USA). The following pre-processing steps were taken: (1) images were corrected for head motion (using MCFLIRT), (2) slice-timing correction was applied, (3) non-brain tissue was removed (using Brain Extraction Tool, BET), (4) functional images were registered to subject-space (T1-weighted structural image) using BBR, (5) this image was registered to MNI152 standard space (FLIRT for linear registration with 12 DOF), (6) high-pass filtering above 0.01 Hz was applied, (7) spatial smoothing was performed at 5 mm full-width half maximum (FWHM), (8) segmentation of gray and white matter was performed using FAST and SIENAX, (9) the first three volumes of each resting-state scan were discarded to achieve field equilibrium, (10) average motion was calculated as the mean of the absolute head movement over all time series for 6 DOF per individual (three translations and three rotations).

**Fig. 1:**
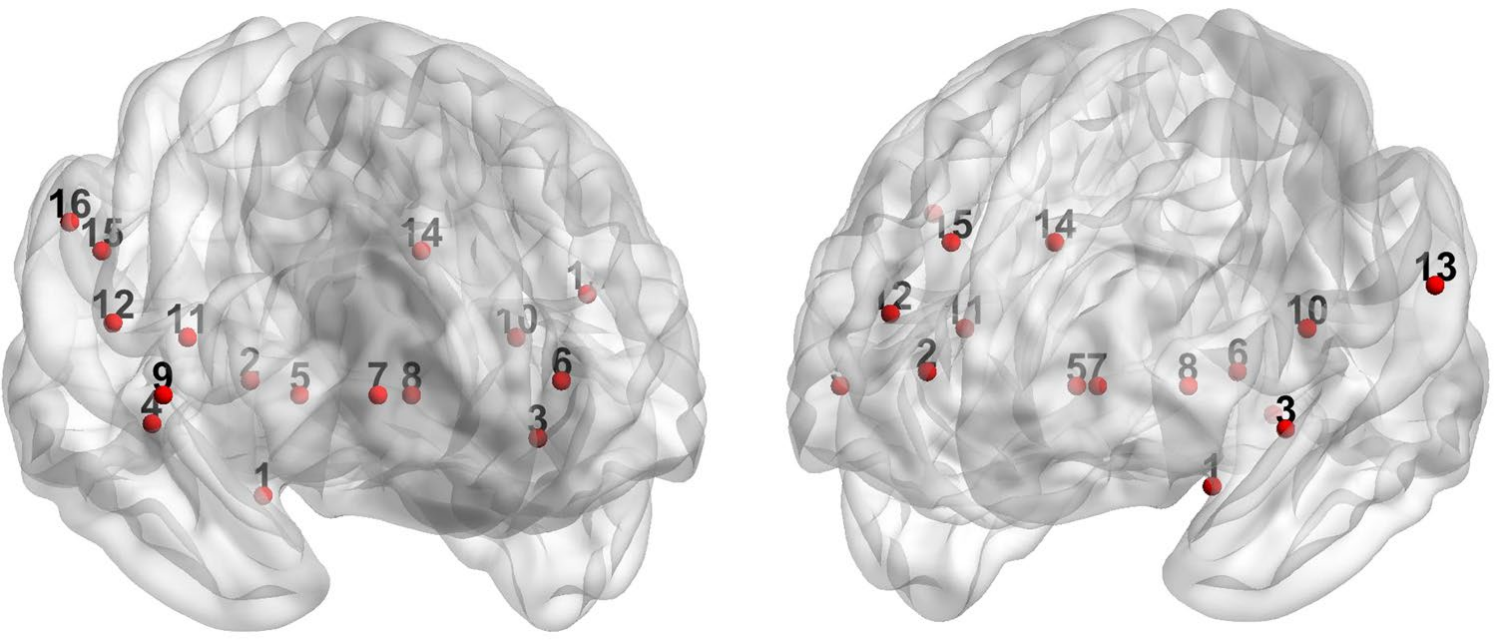
The pain network, based on the positive predictive weights of the NPS (Wager et al. 2013). Areas shown are: 1 = vermis cerebellum; 2 = anterior/mid insula (right); 3 = superior temporal gyrus; 4 = calcarine gyrus; 5 = ventrolateral thalamus (right); 6 = mid insula (left); 7 = hypothalamus; 8 = ventrolateral thalamus (left); 9 = frontal operculum/temporal pole; 10 = dorsal posterior insula/secondary somatosensory area (left); 11 = dorsal posterior insula (right); 12 = somatosensory area (right); 13 = temporoparietal junction; 14 = dorsal anterior cingulate cortex; 15 = supramarginal gyrus; 16 = inferior parietal lobule. BrainNet Viewer version 1.6 was used for visualization (http://www.nitrc.org/projects/bnv/) (Xia et al. 2013)

After preprocessing, a resting-state adjacency matrix (representing an undirected weighted network) was reconstructed per subject as follows. First, time series were scrubbed for motion outliers: time points with frame-to-frame displacement > 1.5 mm (6 DOF) were excluded from further analyses. The remaining time series of chosen atlas regions (see below) were used to calculate the connectivity matrix using Pearson’s correlation coefficients between time series of each pair of regions included. A Fisher transformation on these correlation coefficients was used to get normally distributed correlation values. Atlas regions were based on the atlas of Power et al. (2011). The default mode network (DMN) was constructed from 58 of these nodes (see Power et al. 2011 for all atlas regions and specification of the DMN). To form a pain network, we additionally used a subdivision of the NPS of Wager et al. (2013). In their paper, Wager and colleagues based their NPS on 32 areas (Wager et al. 2013). Sixteen of these areas had positive predictive weights for physical pain, and the other 16 had negative predictive weight. For the current study, only the areas of the NPS with positive predictive weights were used to form a pain network because the interpretation of a network of positive predictive weights is most straightforward, particularly when making comparisons between patient/control groups and medication sessions. See Fig. 1 for a visual overview of this 16-node pain network.

### Network analysis

The resting-state adjacency matrix was used to calculate network topology. We calculated betweenness centrality (BC) and global efficiency (GE) of the resting-state adjacency matrix. BC measures how central a node lies with respect to the rest of the network, and is based on how many shortest paths pass through it. Nodes with high BC represent hubs of the network (Fornito et al. 2016). GE represents efficiency of the underlying network and is a measure of integration. The Brain Connectivity Toolbox (brain-connectivity-toolbox.net) was used to calculate GE per network, and BC of all nodes in the network (with Matlab scripts: efficiency_wei.m and betweenness_wei.m, respectively) (Rubinov and Sporns 2010). BC was then normalized (*z*-scored) per participant, and averaged per network. GE and average BC for all 16 pain nodes represented the pain network, GE and BC for the 58 DMN nodes represented the DMN. For the whole brain (264 nodes), only GE was calculated, since calculating BC (or ‘hubness’) of the whole brain is essentially meaningless.

### Statistical analyses

Mann–Whitney *U* test was performed to investigate the difference between PD and controls on all pain measures, as they were not normally distributed. To investigate the differences in pain and network topology, the independent variable ‘group’ (patients ON and OFF, and controls) and dependent variables BC and GE measures, were entered in multivariate analyses of covariance (MANCOVAs), with average motion during the scan as a covariate. Only differences between patients and controls were considered. To investigate the relationship between pain and network topology, a hierarchical stepwise linear regression was performed per group (controls, PD ON, PD OFF), with the BC and GE of the pain network as independent variables, and clinical pain as dependent variable. In each analysis’ first block, average movement during the scan was added as a covariate to account for motion in the scanner, and a forward stepwise method was utilized to investigate the contribution of each separate independent variable. Scores on the NWC served as a dependent variable. The alpha level was set at 0.05, and tested two-sided.

### Data availability

The datasets generated and analyzed during the current study are available from the corresponding author on reasonable request.

## Results

### Subjects

**Fig. 2:**
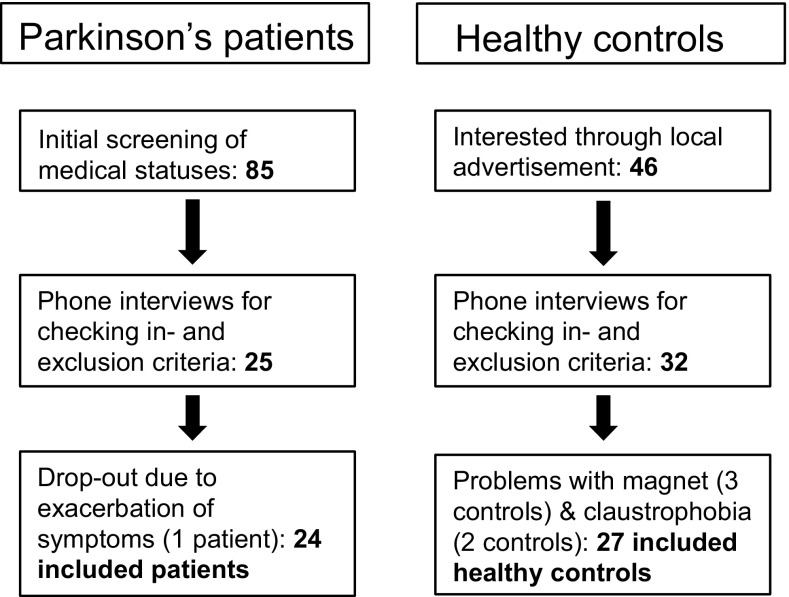
Flowchart of inclusion process

Twenty-four patients and 27 healthy controls participated in this study. See Fig. 2 for an overview of the inclusion process. Patients and controls were matched for age and gender. Education was measured by means of the Verhage-system, ranging from 1 (unfinished lower education) to 7 (finished scientific education) (Verhage 1964). Patients had a lower level of education (*U* = 174.50, *p* = 0.003). Though not significantly different, patients had a slightly lower score on the MoCA [*t* = 1.9 (49), *p* = 0.063]. Patients had a higher score on the Beck’s Depression Inventory (BDI) (Beck et al. 1996), indicating more severe symptoms of depression in patients than in controls (*t* = − 5.107(48), *p* < 0.01). None of the patients experienced dyskinesia during scanning. All participant characteristics are shown in Table 1. Details per patient are shown in Table 2.

**Table 1:** Subject characteristics

	Controls (*n* = 27)	Patients (*n* = 24)	Difference
Age in years (*M*, SD)	59.37 (8.54)	63.42 (7.93)	ns
Education level (*M*, SD)	6.15 (0.86)	5.25 (1.11)	*U* = 174.5, *p* = 0.003
Gender	11 females	7 females	ns
Disease duration in years (*M*, SD)	–	4.08 (3.13)	–
LEDD in mg (*M*, SD)	–	796.29 (616.44)	–
UPDRS during ON phase (*M*, SD)	–	17.67 (7.66)	–
MoCA (*M*, SD)	27.89 (1.89)	26.88 (1.92)	ns
BDI	22.96 (2.24)	30.46 (7.12)	*U* = 82.5, *p* < 0.001

*LEDD* Levodopa equivalent daily dose, *UPDRS* United Parkinson’s disease rating scale, *MoCA* montreal cognitive assessment, *BDI* Beck’s depression inventory

**Table 2:** Overview of clinical characteristics per patient

Patient	Age	Disease duration (years)	LEDD (mg)	Parkinson-medication			Time to scan since medication (h)		UPDRS III (before scan)		Duration of pain (years)	Wearing-off^a^
				Levodopa	DA-agonist	Other	OFF	ON	OFF	ON		
#1	55	3.5	564	Yes			15.0	1.0	24	18.5	3.00	Yes
#2	73	2.0	752	Yes			17.0	1.0	28	20	8.00	Yes
#3	67	10.0	564	Yes			20.5	1.0	20	17	30.00	Yes
#4	72	3.0	375	Yes			16.5	1.5	14	17	3.00	Yes
#5	68	1.0	828	Yes			16.0	2.5	16	11	0.00	No
#6	56	2.0	375	Yes			26.5	2.0	11	7	1.00	No
#7	68	2.0	378	Yes			19.5	5.5	13	13	0.00	Yes
#8	65	8.0	850	Yes		MAO-B inhibitor (rasagiline), COMT inhibitor (entacapone)	12.5	1.5	18	Missing	1.00	Yes
#9	62	4.0	2780	Yes		COMT inhibitor (entacapone)	14.5	3.0	33	18	3.00	No
#10	64	5.5	982	Yes	DA-agonist (pramipexol)		14.5	2.0	25	30	0.25	No
#11	68	2.0	125	Yes			15.5	2.5	6	7	3.00	No
#12	69	1.0	500	Yes			15.0	8.0	19	19	0.00	No
#13	73	5.0	375	Yes			13.5	3.5	37	32	5.00	Yes
#14	70	3.0	1548	Yes		COMT inhibitor (entacapone)	15.5	1.5	17	19	5.00	Yes
#15	71	6.0	1038	Yes	DA-agonist (pramipexol)		8.5	5.5	35	31	7.00	Yes
#16	47	6.0	1428	Yes	DA-agonist (ropinirol)		14.0	1.0	24	9	0.00	No
#17	48	0.5	1000	Yes			15.0	1.5	6	5	0.00	Yes
#18	56	6.0	935	Yes	DA-agonist (pramipexol)	MAO-B inhibitor (rasagiline)	13.0	1.5	31	13	2.00	Yes
#19	66	1.0	90	Yes			16.5	2.0	26	26	5.00	No
#20	53	5.0	615	Yes	DA-agonist (ropinirol)		19.5	1.5	22	18	7.00	Yes
#21	72	13.0	1150	Yes	DA-agonist (pramipexol)	MAO-B inhibitor (rasagiline)	16.0	2.0	23	26	0.00	No
#22	57	1.0	106	No	DA-agonist (pramipexol)		18.5	4.5	18	12	2.00	Yes
#23	51	6.0	1645	Yes	DA-agonist (ropinirol)	Amantadine	14.0	2.5	25	16	5.00	No
#24	61	1.5	108	No	DA-agonist (pramipexol)		27.0	12.0	15	22	0.75	Yes

^a^Wearing off was determined as having 2 or more symptoms that improved with medication-intake, based on (Martinez-Martin and Hernandez 2012). One patient took a combination of an NSAID (Ibuprofen) and acetaminophen on a daily basis, all other patients did not any have pharmacological intervention for their pain

### Pain measures

An overview of types of pain present in patients and controls is shown in Table 3. Pain types are based on classification according to Ford (2010), with ‘Headache’ as an added category. Scores on pain measures were higher in PD patients than in controls (see Table 4). PD patients experienced more chronic pain than controls: 75% of patients had chronic pain compared to 40.7% of healthy controls [Pearson’s *χ*^*2*^(1) = 6.080, *p* = 0.014]. Scores on the NWC were higher in patients (*M* = 11.92, *SD* = 11.77) than in controls (*M* = 4.83, *SD* = 6.03, Mann–Whitney *U* = 412.00, *p* = 0.010).

**Table 3:** Overview of types of pain according to the pain categories of Ford (2010), ‘Headache’ was added as a category

Type of pain	Patients (%)	Controls (%)
Musculoskeletal	16 (66.7%)	9 (33.3%)
Dystonic	3 (12.5%)	0
Neuropathic/radicular	5 (20.8%)	0
Central	0	0
Akathisia	0	0
Headache	0	2 (7.4%)

Multiple types of pain for a single subject were possible

**Table 4:** Differences on pain scores between PD (ON and OFF phase) and healthy controls, tested with Mann–Whitney’s *U* test

	HC versus PD OFF			HC versus PD ON		
	HC (*M*, SD)	PD OFF (*M*, SD)	Difference	Controls (*M*, SD)	PD ON (*M*, SD)	Difference
CAS intensity	4.35 (9.38)	15.87 (22.09)	U = 413.00, *p* = 0.010	4.35 (9.38)	15.91 (18.77)	U = 405.00, *p* = 0.037
CAS affect	2.81 (6.89)	15.74 (22.49)	U = 425.00, *p* = 0.004	2.81 (6.89)	16.78 (20.28)	U = 416.00, *p* = 0.019

*PD* Parkinson’s disease, *HC* Healthy controls, *CAS* colored analogue scale

### Network topology

Next, we investigated the difference between PD patients (PD ON or PD OFF) and controls on network topology (see Table 5). An overview of the findings for each brain network is provided below. GE of the whole-brain network was significantly higher in PD ON versus controls, and showed a trend for higher GE in PD OFF versus controls. BC of the pain network was lower in PD OFF than in controls. GE of the pain network was higher in PD ON than in controls. No differences were found for the DMN, nor were there any differences between patients’ ON and OFF phases.

**Table 5:** Network measures for all groups and networks

Network	HC versus PD OFF			HC versus PD ON		
	HC (*M*, SD)	PD OFF (*M*, SD)	Difference	HC	PD ON	Difference
Whole brain						
GE	125.31 (60.26)	159.38 (44.05)	*F*(1, 48) = 3.80; *p* = 0.057	125.31 (60.26)	175.86 (72.24)	*F*(1, 48) = 8.93; *p* = 0.004
DMN						
BC	0.02 (0.12)	− 0.01 (0.15)	ns	0.02 (0.12)	− 0.001 (0.13)	ns
GE	0.30 (0.33)	0.37 (0.34)	ns	0.30 (0.33)	0.38 (0.25)	ns
Pain						
BC	0.11 (0.23)	− 0.04 (0.26)	*F*(1, 48) = 4.70; *p* = 0.035	0.11 (0.23)	0.02 (0.30)	ns
GE	11.40 (10.61)	23.74 (37.63)	ns	11.40 (10.61)	20.81 (21.30)	*F*(1, 48) = 4.40; *p* = 0.041

MANCOVAs were performed, with average motion during the scan as a covariate

*BC* betweenness centrality, *GE* global efficiency, *DMN* default mode network, *PD* Parkinson’s disease, *HC* healthy controls, *ns* not significant

### Relationship between pain and network topology

**Fig. 3:**
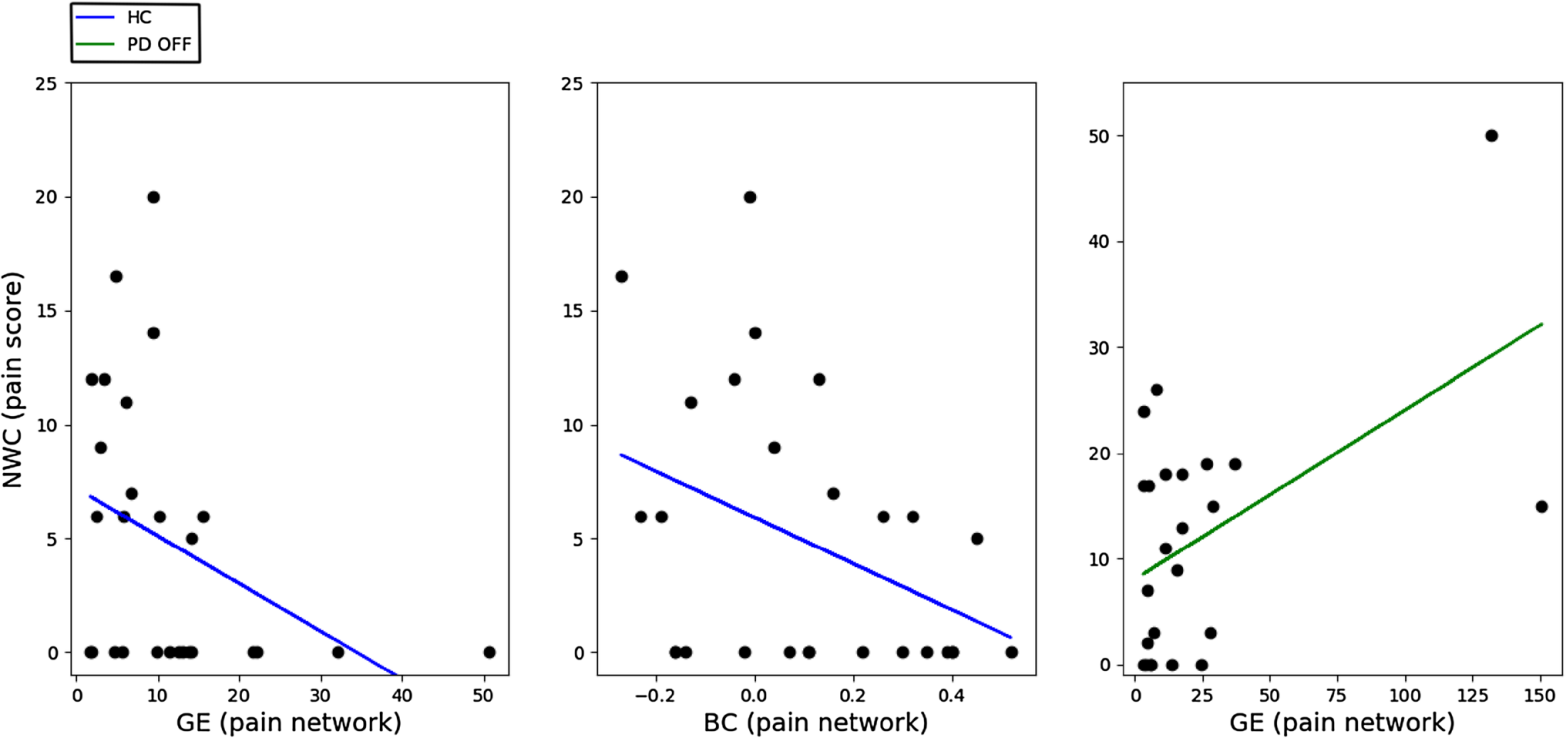
Plots of separate significant effects of the regression analyses. Left panel (blue): a negative linear association of GE of the pain network with NWC-scores for HC. Middle panel (blue): a negative linear association of BC of the pain network with NWC-scores for HC. Right panel (green): A positive linear association of GE of the pain network with NWC-scores for PD OFF. *GE* global efficiency, *NWC* number of words chosen, *BC* betweenness centrality, *PD* Parkinson’s disease, *HC* healthy controls, *PD OFF* PD patients during OFF phase

We performed three regression analyses to investigate the relationship between pain and topology of the pain network, i.e., one within each group. As a control analysis, we investigated whether LEDD and functional topology of the pain network were related. During ON, neither BC (*r* = − 0.081, *p* = 0.707), nor GE (*r* = 0.298, *p* = 0.158) were related to LEDD. Additionally, during OFF, neither BC (*r* = − 0.067, *p* = 0.754), nor GE (*r* = − 0.213, *p* = 0.318) were related to LEDD. Scores on the NWC were also not related to LEDD (*r* = 0.102, *p* = 0.634). Table 6 lists the parameters of all three regression analyses. Since we used a forward stepwise method, only significant predictors in addition to the entered covariate are shown. In controls, the final model (Step 3) contained BC and GE of the pain network as predictors for the NWC-scores [*R*^2^ = 0.29, *F*(3) = 3.14, *p* = 0.045]. In PD OFF, the final model (Step 2) contained GE of the pain network as a predictor for NWC-scores [*R*^2^ = 0.29, *F*(2) = 4.22, *p* = 0.029]. In PD ON, none of the predictors were significant predictors for scores on the NWC. For clarity, plots of the regression slopes are shown in Fig. 3, one for each significant predictor.

**Table 6:** One linear hierarchical regression was performed for each group (controls, PD ON and PD OFF medication)

Group	Step	Independent variables	Unstandardized B	Std. error of B	Standardized B	*p* value
HC	Step 1	Average motion	− 0.055	18.62	− 0.001	ns
	Step 2	Average motion	0.203	17.50	0.002	ns
		BC of pain network	− 10.17	4.89	− 0.39	0.049
	Step 3	Average motion	− 8.65	16.88	− 0.09	ns
		BC of pain network	− 9.99	4.57	− 0.38	0.039
		GE of pain network	− 0.218	0.10	− 0.38	0.046
PD OFF	Step 1	Average motion	− 5.43	65.11	− 0.02	ns
	Step 2	Average motion	− 38.30	57.43	− 0.13	ns
		GE of pain network	0.171	0.059	0.55	0.009
PD ON	Step 1	Average motion	27.32	36.34	0.158	ns

To control for motion, average motion during scanning was entered at Step 1 as a covariate, after which a forward stepwise method was used to add significant independent variables to the model

*BC* betweenness centrality, *GE* global efficiency, *ns* not significant

## Discussion

This study investigated both pain and functional network topology, as well as their interrelatedness in PD. Pain as a symptom of PD has been investigated before, and our results corroborate the increased pain experience in PD compared to controls (Broen et al. 2012; Fil et al. 2013). Pain scores in our patient group were not different when they were on or off medication. Although dopamine could have an ameliorating effect on subjective pain experience (Hagelberg et al. 2004; Brefel-Courbon et al. 2005), this effect has not consistently been found (Flores et al. 2004; Dellapina et al. 2011; Polli et al. 2016).

Our results on functional network topology indicate that PD patients had a higher efficiency of the whole brain off medication (trend-level) as well as on medication (significance-level) compared to healthy controls. Global efficiency of the pain network was also higher compared to controls, but only when PD patients were on medication. Compared to controls, PD patients’ pain network had a lower hubness (BC) when off medication. These results are not in line with previous findings relating to topology of resting-state networks: a decrease in global efficiency of the whole brain network (Skidmore et al. 2011) and a decrease in efficiency of the cortico-basal ganglia motor circuit (Wei et al. 2014) in PD patients off medication has been reported using fMRI. Olde Dubbelink and colleagues show that global efficiency of the functional network assessed with MEG is dynamic across the disease course: de novo patients have global efficiency similar to controls, but global efficiency and local integration decrease with disease progression (Olde Dubbelink et al. 2014). Our results suggest an increase, not a decrease in (whole brain) efficiency in PD. This could point towards a dynamic mechanism of hub-overload: brain disorders, such as AD and schizophrenia, have recently been shown to preferentially affect highly connected hub regions. Certain hubs appear to become overloaded, after which a neural traffic rerouting occurs from the overloaded (provincial) hubs towards connector hubs (Stam 2014). This process of shifting loads in the hierarchy continues until the most centrally situated hubs are overloaded and the disease has reached a chronic phase. This theory was partly confirmed in Alzheimer’s disease (AD) (Jones et al. 2016), where hub-overload was found before any clinical symptoms became apparent. Similar results were found in glioma and multiple sclerosis patients, where connectivity between hubs and non-hubs was higher in patients at diagnosis than in controls (Derks et al. 2017; Meijer et al. 2017). Hub-overload could, therefore, be a central issue in these brain disorders. Applying this theory to PD as a dynamic, progressive disease, our current findings might be understood as follows: patients were in a relatively early stage of the disease, with relatively few symptoms. The reported increase in global efficiency could, therefore, reflect the early relaying of information. As the disease progresses, such rerouting of connectivity may lead to an overload of the hubs of the brain, which will hypothetically be accompanied by increasing symptom burden. Subsequently, a breakdown of the system could result in further progression of the disease.

We found that higher global efficiency of the pain network was associated with more clinical pain in PD patients off medication. Global efficiency and betweenness centrality were associated with less pain in healthy controls. These findings may indicate group differences as to how pain is reflected in the pain network. Higher global efficiency could indicate a more integrated network (Stam 2014), or a network in which information can be processed in parallel (Skidmore et al. 2011). Regarding our results on the group of healthy controls this would signify a quickly transferred signal within the pain network, which reflects little clinical pain over the last month. Additionally, when hubness of the pain network is higher, controls experience less clinical pain. Alternatively, it could suggest an ongoing pathological process: increased efficiency advocates spreading of seizures in epilepsy patients (DeSalvo et al. 2014). Higher efficiency of the pain network in PD could, therefore, also be seen as pathological, with pain as a clinical manifestation in PD patients. More research is needed to replicate our results and investigate causality of either increased or decreased efficiency and centrality. It should be noted that two of our included patients had an exceptionally high GE of the pain network. One patient had painful polyneuropathy, the other had dystonia, which caused pain. After careful consideration, we chose to keep these patients in the dataset, as we could not determine that these high values of GE were due to artifacts or other technical issues. Instead, we conclude that these subjects represent variation of GE in PD patients with pain.

Even though this study provides insight into a possible underlying mechanism of pain in PD, several issues should be considered. Our study focused on neural processing of pain, and did not take any other clinical factors into account that might influence pain. One such factor is the presence of symptoms of depression, which is known to influence pain experience (Rana et al. 2017). Other examples are age, gender, disease duration, and severity of motor symptoms, as well as a possible effect of wearing-off, the loss of response to dopaminergic medication over time. The effect of wearing-off is the quick re-emergence of symptoms after medication-intake. Even though our patients had relatively short disease duration, more than half experienced wearing off (see Table 2). In addition, the effect of pharmacological subclasses of PD medication could be of influence. Due to the complexity of dopamine-receptor binding for the various subclasses of dopamine medication, this could not be analyzed within the small sample of the current study. Another issue regarding PD medication is the time from last medication-intake until MRI-scanning, specifically during the ON phase: an average of 2.9 h remained between medication-intake and the resting-state scan. Since the half-life of levodopa-medication is 1–2 h, this could have influenced our results, and we can, therefore, not be absolutely certain that patients were truly in the ON phase. To find independent contributions of all aforementioned factors in the context of pain and the functional brain network, a larger group of patients is needed, preferably representing both low and high pain phenotypes. Future research that includes patients with high pain intensity could also reinforce our results. In this study, we investigated pain as a continuous variable, including subjects with a score of 0. A continuous scale was chosen, as we cannot be sure at which point pain becomes a pathological symptom of PD. A score of 0 might, therefore, also represent a pathological processing of pain. Moreover, the pain network as we included it might not explain all of patients’ pain experience: we chose to include only positive predictive weights of the NPS (Wager et al. 2013), and might, therefore, have missed information coming from the subdivision of the NPS of the negative predictive weights. In addition to this, our pain network did not include any areas typical of the brainstem descending modulatory network (including the periaqueductal gray and the rostral ventral medulla), which modulates pain further by facilitation or inhibition at the spinal level (Vanegas and Schaible 2004; Tracey and Mantyh 2007). Finally, most neural activation studies into pain are based on groups of healthy and relatively young volunteers, who receive acute pain stimuli. The generalization towards clinical pain in a patient group might, therefore, reflect a different mechanism as compared to other studies into clinical pain.

To our knowledge, this is the first study that investigates resting-state functional connectivity-derived network measures in PD to study pain. Our main findings are a higher integration of the whole brain in PD patients compared to controls, a higher integration of the pain network in PD patients (on medication) compared to controls, and a lower hubness of the pain network in patients (off medication) compared to controls. Additionally, a positive association was found between clinical pain and hubness of the pain network in patients off medication, but because of low sample size and statistical outliers, these results should be interpreted cautiously. Further (replication) studies are needed to substantiate the nuances of network measures, and to investigate other symptoms to gain insight in the entirety of the disease.
